# Actual patient position versus safety models: Specific Absorption Rate implications of initial head position for Ultrahigh Field Magnetic Resonance Imaging

**DOI:** 10.1002/nbm.4876

**Published:** 2022-12-12

**Authors:** Emre Kopanoglu

**Affiliations:** ^1^ Cardiff University Brain Research Imaging Centre (CUBRIC), School of Psychology Cardiff University Cardiff UK

**Keywords:** initial patient position, magnetic resonance imaging, parallel‐transmit, patient safety, RF pulse design, specific absorption rate, ultrahigh field MRI

## Abstract

Specific absorption rate (SAR) relates power absorption to tissue heating, and therefore is used as a safety constraint in magnetic resonance imaging (MRI). This study investigates the implications of initial head positioning on local and whole‐head SAR. A virtual body model was simulated at 161 positions inside an eight‐channel parallel‐transmit (pTx) array. On‐axis displacements and rotations of up to 20 mm/degrees and off‐axis axial/coronal translations were investigated. Single‐channel, radiofrequency (RF) shimming (i.e., single‐spoke pTx) and multispoke pTx pulses were designed for seven axial, five coronal and five sagittal slices at each position (the slices were consistent across all positions). Whole‐head and local SAR were calculated using safety models consisting of a single (centred) body position, multiple representative positions and all simulated body positions. Positional mismatches between safety models and actual positions cause SAR underestimation. For axial imaging, the actual peak local SAR was up to 4.2‐fold higher for both single‐channel and 5‐spoke pTx, 3.5‐fold higher for 3‐/4‐spoke pTx, and 2‐fold higher for RF shimming and 2‐spoke pTx, compared with that calculated using the centred body position. For sagittal and coronal imaging, the underestimation of peak local SAR was up to 5.2‐fold and 3.8‐fold, respectively. Using all body positions to estimate SAR prevented SAR underestimation but yielded up to 11‐fold SAR overestimation for RF shimming. Local SAR of single‐channel and pTx multispoke pulses showed considerable dependence on the initial patient position. RF shimming yielded much lower sensitivity to positional mismatches for axial imaging but not for sagittal and coronal imaging. This was deemed attributable to the higher degrees‐of‐freedom of control offered by the investigated coil array for axial imaging. Whole‐head SAR is less sensitive to positional mismatches compared with local SAR. Nevertheless, whole‐head SAR increased by up to 80% for sagittal imaging. Local and whole‐head SAR were observed to be more sensitive to positional mismatches in the axial plane, because of larger variations in coil‐tissue proximity. Using all possible body positions in the safety model may become substantially over‐conservative and limit imaging performance, especially for the RF shimming mode for axial imaging.

## Graphical abstract

A positional mismatch between the actual head position and that of the safety model leads to underestimation of the specific absorption rate, by up to 4.2‐fold for peak local SAR and 1.4‐fold for whole‐head SAR. RF shimming offers reduced sensitivity to positional variations compared with single‐channel transmit and parallel transmit, for axial imaging.

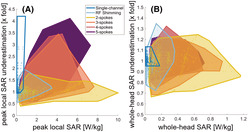

**Abbreviations used**
MRI: magnetic resonance imaging
psSAR: peak spatial value of local SAR
pTx: parallel‐transmit
R/L/A/P: right/left/anterior/posterior
RF: radiofrequency
SAR: specific absorption rate
UHF‐MRI: ultrahigh field MRI
VOPs: virtual observation points
whSAR: whole‐head SAR

## 1 INTRODUCTION

Despite the benefits of ultrahigh field (UHF) magnetic resonance imaging (MRI),^1^, ^2^, ^3^ the wavelength becomes comparable to body dimensions. This leads to wavelength artefacts and causes artificial contrast variations in acquired images.^4^ To mitigate these contrast variations, which are excitation related, parallel‐transmit (pTx) arrays and tailored pulses are commonly used.^5^, ^6^, ^7^, ^8^, ^9^, ^10^ The independent control of multiple transmit coils increases the variability in the local variation of the specific absorption rate (SAR, local SAR). This has caused concerns over inadvertently causing regions of very high local SAR, especially compared with the volume coils, which offer a spatially balanced distribution of power around coil conductors. Moreover, local SAR may reach its limits even when global SAR is under its safety limits.^11^, ^12^, ^13^, ^14^ Hence, local temperature and local SAR^13^, ^15^ have been proposed as safety limits in pulse design.^16^, ^17^, ^18^, ^19^, ^20^

In MRI, the interaction between tissues and radiofrequency (RF) coil elements leads to RF power absorption in tissues. These interactions can be summarised in the so‐called Q‐matrix,^21^, ^22^ which can be compressed using virtual observation points (VOPs) to accelerate computations.^23^ Q‐matrices and VOPs have been used to constrain SAR offline^20^ and in real time.^24^ While Q‐matrices or VOPs can be used to ensure adherence to safety limits,^25^, ^26^ simulations may not accurately reflect the actual scan. Therefore, previous studies proposed using safety margins to account for modelling errors,^27^ intersubject variability^15^, ^28^, ^29^ and hardware uncertainties.^30^, ^31^

One of the scan variables that local SAR depends on is the patient position. Patient position is affected by many variables, which for head MRI include the shape and size of the head, variations in padding material choices within and across scanners, and circumstantial adjustments such as using extra padding to reduce discomfort or the patient settling in. While body position varies between patients, in practice, safety models (Q‐matrices/VOPs) provided with RF coils and MRI scanners are fixed (i.e., not position dependent). This leads to a mismatch between the actual interaction between fields and tissues and the assumed interaction. If a safety model contains a single body position centred inside the coil structure, this might not be representative of an off‐centre patient position and may be under‐conservative. Alternatively, if a safety model contains all possible positions, then it may be over‐conservative for a position approximately equidistant to all coil elements. Previous studies have investigated the effect of within‐scan patient motion^14^, ^32^ and/or patient position on SAR at 1.5T,^33^, ^34^ 3T,^35^, ^36^ 7T^28^, ^37^, ^38^ and 9.4T^39^ for adult models and neonates,^40^, ^41^ with several studies reporting upwards of 2‐fold variation because of motion/positional variations. However, most of these studies focused on different subsets of the six degrees‐of‐freedom of motion and random pulses or RF shimming. Although Kopanoglu et al.^14^ investigated all six degrees‐of‐freedom of motion, they focused on the effect of within‐scan patient motion rather than a mismatch between the initial patient position and the safety model. Hence, the effect of all six degrees‐of‐freedom of initial positional variations on local SAR has not yet been investigated for practical pTx pulses and contrasted against single‐channel operation of a coil structure.

This study investigates the implications of initial patient position on local and whole‐head SAR. For this purpose, a virtual body model was simulated inside an eight‐channel transmit coil at 161 positions. All six degrees‐of‐freedom of positional variations were included by displacing/rotating the coil by up to 20 mm/degrees relative to the body model. Off‐axis displacements in axial and coronal planes were also investigated. Single‐channel, 1‐/2‐/3‐/4‐/5‐spoke pTx pulses were designed for seven axial slices at each position. Whole‐head and 10‐g‐averaged local SAR calculated using the actual body position were compared with values calculated using safety models that consisted of the centred body position, and five and nine body positions displaced in the axial plane. Whole‐head and local SAR were also calculated using a safety model that consisted of all simulated positions inside the coil for selected pulses. Pulses were also designed for five sagittal and five coronal slices for two different definitions of region‐of‐interest in each case. This study builds on two International Society for Magnetic Resonance in Medicine (ISMRM) conference abstracts.^42^, ^43^

## 2 METHODS

### 2.1 Electromagnetic modelling

**FIGURE 1:**
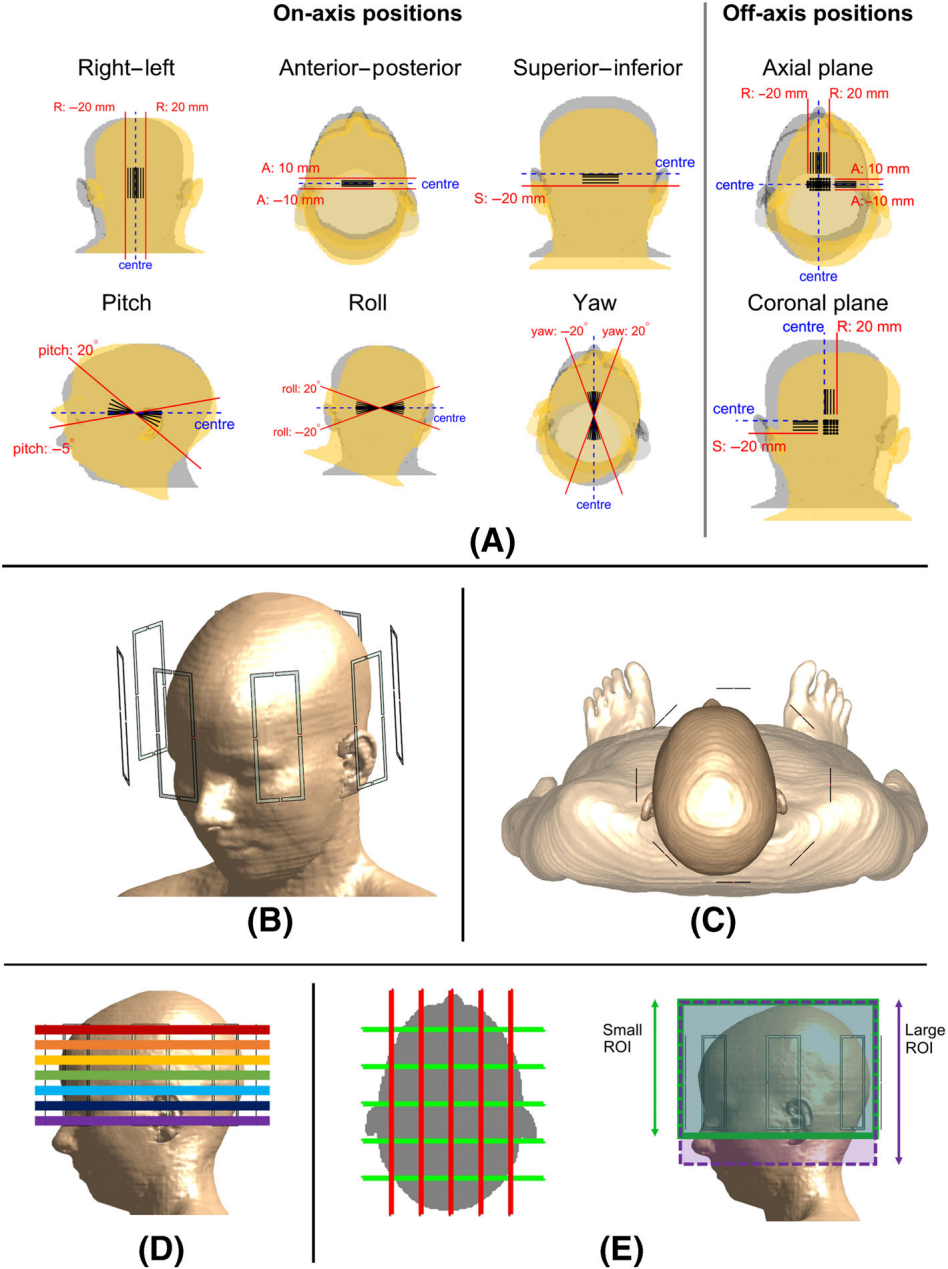
The body model Ella was simulated inside a generic eight‐channel coil. (A) Simulations were carried out at 161 positions including on‐axis displacements and rotations along and around the three Cartesian axes, and off‐axis displacements in the axial and coronal axes. Markers and lines indicate how the body model is displaced with respect to the coil, with extreme cases shown in red. Grey and yellow shaded models show the body at the centre and the furthest off‐centre position. (B) The coil array consisted of eight loop elements. The computation domain included the shoulders (omitted in this view). (C) The body is shown in location Left: 15 mm, Posterior: 5 mm. (D and E) RF excitation pulses were designed for (D) Seven axial slices and (E) Five coronal and five sagittal slices (slice thickness not to scale) at all positions. A small and a large target region of interest (ROI) was defined for sagittal and coronal pulses. Whole‐head SAR (whSAR) was calculated over the volume of the tissue visible in (D). RF, radiofrequency; SAR, specific absorption rate

Electromagnetic simulations were performed using Sim4Life (Zurich MedTech AG, Zurich, Switzerland) with details following previously published methods^14^ for continuity and comparability. The virtual body model Ella (version v. 3.1)^44^ was simulated at 161 different relative positions with respect to the coil structure, including displacements and rotations of up to 20 mm/degrees. Fifty‐seven off‐centre positions were 1, ± 2, ± 5, ± 10, ± 15 and ± 20 mm towards right; 1, ± 2, ± 5 and ± 10 mm towards posterior; 1, 2, 5, 10, 15 and 20 mm towards inferior, ± 1°, ± 2°, ± 5°, 10°, 15° and 20° in pitch; ± 1°, ± 2°, ± 5°, ± 10°, ± 15° and ± 20° in roll; and ± 1°, ± 2°, ± 5°, ± 10°, ± 15° and ± 20° in yaw (Figure 1A). A further 103 off‐axis positions were generated by changing the relative position of the model towards both right and inferior, and both right and posterior, using a combination of the given values. Values were limited in anterior and pitch, and combinations of ± 10 mm in anterior and 20 mm towards left were excluded to avoid an overlap between coil elements and the model. The reported values are the relative position of the body model with respect to the coil structure, with positive rotation values denoting clockwise rotation around left for pitch, anterior for roll and superior for yaw.

An eight‐channel generic coil structure was modelled (inner diameter: 230 mm). The coil was designed such that coil elements 1 and 5 would be parallel to the patient table (excluded in the simulations). Because the body model's head is at a slight angle with respect to the surface that would align with the back of the model, it is also at a slight angle with respect to the coil elements. The aligning of the body model was performed manually. At the reference position (hereby onwards referred to as the centred position), the minimum distance between the head and the closest coil elements was 16, 34, 17 and 47 mm on the posterior, left, anterior and right sides, respectively. Figure 1B,C show the simulated structure for the centred body position (Figure 1B) and the off‐centre body position of left (15 mm) and posterior (5 mm) (Figure 1C). Each loop coil had 40 mm width, 110 mm height, 3 mm microstrip width, and four slots: one was used as the feed port and three for capacitors. For the centred body position, the coil elements were tuned to 295 MHz using 4.2‐pF capacitors in each slot. The average input resistance across the coil elements was measured to be 6 Ω, which was assigned as the output resistance of the feed ports. This yielded a return loss of less than −10 dB across all ports (centred position). Tuning capacitor and port resistance values were unaltered across positions, inherently incorporating changes in coil loading and coil matching because of positional variations. Input power (i.e., total available power minus total reflected power) at each feed port was normalised to 1 W. This overrides any imperfections in coil matching at the feed ports and simulates a feedback circuitry that keeps the input power level consistent. This normalisation also ensures that equal power is transmitted through each coil for the quadrature (single‐channel) mode, as will be detailed later in the Methods section. The effect of this normalisation was observed to vary less than 8% across all coils, considering the centred location and the location with the largest displacement (right [R]: 20/anterior [A]: 10 mm).

Moving any structure within the computational space may affect voxelisation. Specifically for the body model, this could affect how tissues smaller than the voxel size are discretised and may lead to inconsistencies across cases. Furthermore, registering the fields of different positions of the body model would require three‐dimensional interpolation. To avoid both potential sources of error, the coil structure was displaced instead of the body model. To minimise the effect of voxelisation on the coil structure, an automatic high‐resolution adaptive voxelisation with 1 mm maximum step size was enforced on the coil elements. A finer resolution was tested at selected positions but the effect on field distributions was negligible.

Inside the computation domain, 46 different tissues/organs were simulated (Figure 1B), including the cerebrospinal fluid and the shoulders, as recommended.^45^ The local SAR at the edge of the computation domain was verified to be always at least 30 dB lower than the spatial maximum. The body model was discretised using an isotropic resolution of 2 mm. The fields were manually compared to ensure a smooth variation across positions. The coil elements were displaced automatically; however, the models were checked manually for connectivity and voxelisation issues. The number of voxels increased for rotation cases to ensure connectivity, yielding between 6.5 million and 30 million voxels across model positions.

### 2.2 Pulse design and SAR calculations

Field and tissue density data were mapped onto a predefined grid that encloses the head (size: 180 x 215 x 250 mm) in Sim4Life to ensure consistent voxelisation before those were exported to Matlab (Mathworks Inc., Natick, MA, USA) for pulse design and evaluations. Small‐tip angle^46^ slice‐selective pTx spoke pulses^47^ were designed using an adaptation of the Matching Pursuit‐guided Conjugate Gradient algorithm.^48^ The normalised root‐mean‐squared error in the excitation profile and the RF power were calculated using an L2‐norm. The cost function was defined as the sum of the two terms, the latter regularised by a Tikhonov parameter λ=0.5. ^47^ Candidate spokes were defined on a 11 x 11 k‐space grid with $\delta k_{x}=\delta k_{y}=4\;m^{-1}$. The $k_{x}=k_{y}=0$ spoke was enforced as the first spoke. Spokes were added iteratively until a predefined number of spokes ( $N_{s}$) was reached. In each iteration, the algorithm added a candidate spoke to the set of selected spokes, optimising the coefficients for all spokes and channels using Conjugate Gradient Descent.^49^ The candidate spoke that minimised the cost function was selected using Matching Pursuit.^50^ Finally, the coefficients for each spoke and channel were reoptimised using the updated set of spokes while relaxing the phase of the target profile.^51^ Omitted details of the algorithm can be found in a previous study.^52^ Coefficients were converted to slice‐selective RF pulse waveforms using time‐optimal trapezoidal gradient waveforms^53^ and sinc pulse envelopes.^54^ Next, 1‐/2‐/3‐/4‐/5‐spoke pulses were designed at each position; 1‐spoke pulses are equivalent to RF shimming with both magnitude and phase optimisation. The single‐channel transmit case was also investigated by combining the channels using the quadrature excitation mode (i.e., equal input power in each coil element with a progressive phase increment of π/4 across channels) and scaling the channel weights to achieve the target average flip angle. Pulse design parameters were borrowed from Kopanoglu et al.,^14^ which closely followed the ISMRM RF Pulse Design Challenge.^55^ Design parameters were: number of coils ( $N_{c}$) = 8; flip angle = $30^{\circ }$; excitation field of view (aligned to the body model at each position) = 180×215×250 mm; excitation matrix size = 122×151×140; and slice separation ( Δz) = 18 mm.

Local SAR calculations were performed using voxel‐wise Q‐matrices^22^ via

$$Q_{\mathrm{ij}}\left(r\right)=\frac{1}{2\rho \left(r\right)}\left[J_{x,j}^{H}E_{x,i}+J_{y,j}^{H}E_{y,i}+J_{z,j}^{H}E_{z,i}\right],$$

where $\rho \left(r\right)$ is the tissue mass density (kg/m^3^), $E_{u,v}$ (V/m) and $J_{u,v}$ (A/m^2^) are complex electric field and current density, respectively, along axes u=x, y or z with v=i or j being the index of the transmit channel, and the superscript H denotes Hermitian conjugation. Entries of the Q‐matrix were then averaged over 10 g of tissue with cubical volumes.^56^ Whole‐head SAR (whSAR) was calculated over a volume of $4.02\times 10^{-3}$ m^3^ (shown region in Figure 1D) and a mass of 4.4 kg. The peak spatial value of local SAR (psSAR) and whSAR are reported.

### 2.3 The effect of number of positions and orientations in safety models on SAR calculations

Five different SAR modelling approaches were investigated. For these comparisons, single‐channel and 1‐/2‐/3‐/4‐/5‐spoke pTx pulses were designed for seven axial slices from cerebellum to crown (Figure 1D) at each head position, yielding a total of 1127 single‐transmit and 5635 pTx pulses. For each pulse, local SAR was calculated using the Q‐matrix of the body model at that position, denoted by $\mathrm{SA}R_{\text{actual}}$.

Case I Centred safety model: for each pulse, local SAR was calculated using the Q‐matrix of the centred body position ( $\mathrm{SA}R_{\text{centre}}$). The underestimation factor ( $\mathrm{SA}R_{\text{actual}}/\mathrm{SA}R_{\text{centre}}$) represents positional mismatch on a scanner with a fixed safety model because the pulse is designed using position‐dependent B_1_^+^‐maps while the Q‐matrix belongs to the centred position. By using $\text{psSA}R_{\text{centre}}$ as a threshold for ${\mathrm{SAR}}_{\text{actual}}$, the size of the region that was exposed to higher local SAR than estimated was demonstrated and is reported in cubic centimetres.

Case II More representative safety models: safety models on scanners can also be generated by combining multiple Q‐matrices belonging to different positions. First, local SAR was calculated using Q‐matrices from five positions, which were centre and ± 5 mm shifts along anterior–posterior and right–left ( $\mathrm{SA}R_{5\text{positions}}$). Then the safety model was expanded to nine positions by also including ± 10 mm shifts along anterior–posterior and right–left ( $\mathrm{SA}R_{9\text{positions}}$). The underestimation factors $\mathrm{SA}R_{\text{actual}}/\mathrm{SA}R_{5\text{positions}}$ and $\mathrm{SA}R_{\text{actual}}/\mathrm{SA}R_{9\text{positions}}$ are reported.

Case III Safety model that includes all the simulated positions: a more inclusive safety model in terms of head positions reduces SAR underestimation. Consequently, a potential approach is to use all possible head positions inside the coil, which means that SAR is never underestimated but may be overestimated. The SAR calculated using the safety model that consisted of all 161 positions is denoted as $\mathrm{SA}R_{\text{eachpos}}$. The 175 pTx pulses designed for centre and all combinations of right = ± 10 mm and anterior = ± 10 mm, were evaluated. For this case, overestimation is reported instead of underestimation, calculated as $\mathrm{SA}R_{\text{eachpos}}/\mathrm{SA}R_{\text{actual}}$. Note that these 161 positions may not necessarily be an exhaustive set of all possible head positions inside the coil, and pulses at the positions closest to the coils were not selected for evaluation.

psSAR and whSAR values are reported for all 6762 pulses. Pulse duration has a direct effect on SAR,^57^ with longer pulses having reduced SAR because they have more time to excite the same flip angle. Therefore, two comparisons were provided. First, time‐normalised SAR, which normalises all pulses to the same pulse duration, regardless of the number of spokes that was used.^57^ The common pulse duration was determined such that the highest psSAR was less than 10 W/kg and the highest whSAR was less than 3.2 W/kg across all pulses. Second, variations in the two metrics (psSAR and whSAR) were compared with each other across positions. The duration of each pulse was individually adjusted so that its whSAR was 3.2 W/kg, and the psSAR values are reported.

Where reported, statistical analyses were performed using a paired *t*‐test with a significance level of *p* = 0.05.

### 2.4 Effect of slice orientation

Single‐channel and 1‐/2‐/3‐/4‐/5‐spoke pTx pulses were also designed for five sagittal and five coronal slices (Figure 1E), with two different target regions of interest (ROIs), yielding a further 19,320 pulses. The tissues outside the ROIs were defined as do‐not‐care regions, where no constraints were imposed on the flip angle. Calculations for Case I were repeated (i.e., $\mathrm{SA}R_{\text{actual}}/\mathrm{SA}R_{\text{centre}}$) to investigate the effect of slice orientation on SAR underestimation. Both peak local and whole‐head SAR are reported.

### 2.5 Variation of patient head position within commercially available head coils

The variation of the head position was investigated within an eight‐channel transmit/32‐channel receive and a single‐channel transmit/32‐channel receive head coil (both Nova Medical, MA, USA) used within a 7‐T Siemens Magnetom scanner (Siemens Healthineers, Erlangen, Germany). The inner dimensions of the receive array were 205 mm (anterior–posterior) by 185 mm (right–left) for both coils. Local ethics committee approval and written informed consent from each participant were acquired. Healthy participants were imaged using a three‐dimensional gradient echo sequence, at a total of 66 positions for the pTx coil and 48 positions for the single‐channel coil. After imaging data were acquired, each participant was asked to move their head to a different position that they felt comfortable in, and the process was repeated. All images were acquired within a field of view that was fixed in scanner coordinates. Maximum intensity projections were taken along the superior–inferior direction, after which the leftmost/rightmost/posteriormost/anteriormost extent of the head was determined using thresholding at 20% of the maximum intensity. The extent in the four Cartesian directions includes the effect of rotations as well.

Head positions were also investigated across seven datasets containing 102 participants (made available through OpenNeuro^58^, ^59^, ^60^, ^61^, ^62^, ^63^, ^64^) scanned at 3 T.

These investigations were undertaken to provide an understanding of possible positional variations in practice. However, neither the 7T pTx and single‐channel coils, nor the coils in studies shared in the OpenNeuro website, bear resemblance to the coil structure simulated in the current study, and therefore do not have any direct implications for the results presented here.

## 3 RESULTS

**FIGURE 2:**
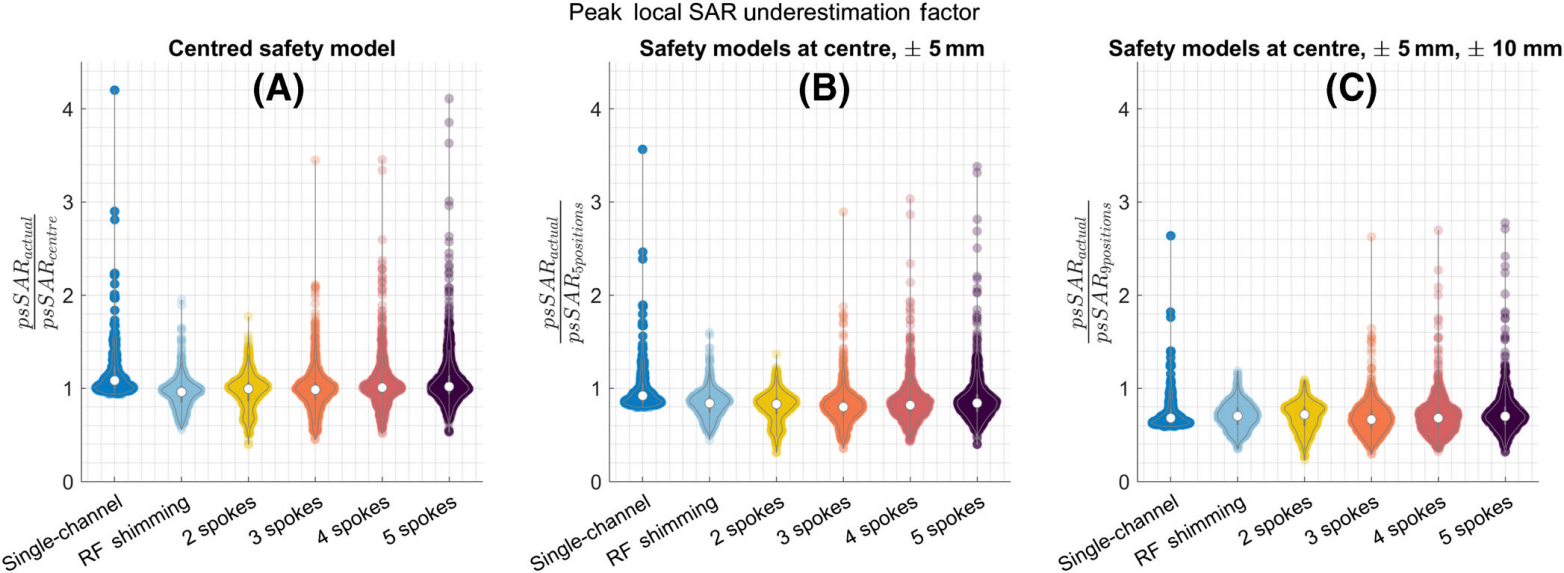
Violin plots show the underestimation factor of the peak local SAR when the safety model consists of (A) The centred body position, (B) Five positions (centre, Right: ±5 mm, Anterior: ±5 mm) and (C) Nine positions (centre, Right: ± 5 and ±10 mm, Anterior: ±5 and ±10 mm). Pulses designed for all 161 positions were evaluated. psSAR, peak spatial value of local SAR; RF, radiofrequency; SAR, specific absorption rate

Using a centred and fixed body model yielded a considerable underestimation of the peak local SAR. The actual peak local SAR was up to 4.2‐fold higher than that estimated by the centred body model, for both single‐channel and pTx. For pTx, the sensitivity of local SAR calculations to positional mismatches increased as the complexity of the pulse increased. RF shimming and 2‐spoke pulses showed the least sensitivity to initial patient positioning across all cases, including single‐channel transmit. Compared with the estimated peak local SAR using the centred model, the actual psSAR was up to 2.0‐fold higher for RF shimming (1‐spoke), 1.8‐fold higher for 2‐spoke pulses, 3.5‐fold higher for 3‐/4‐spoke pulses and 4.2‐fold higher for 5‐spoke pulses (Figure 2A).

Using more body positions reduced the sensitivity of local SAR to positional mismatch. Nevertheless, the actual psSAR was still higher than the estimated maximum by up to 3.6‐ and 3.4‐fold for the five‐position case and 2.7‐ and 2.8‐fold for the nine‐position case, for single‐channel and pTx, respectively.

**FIGURE 3:**
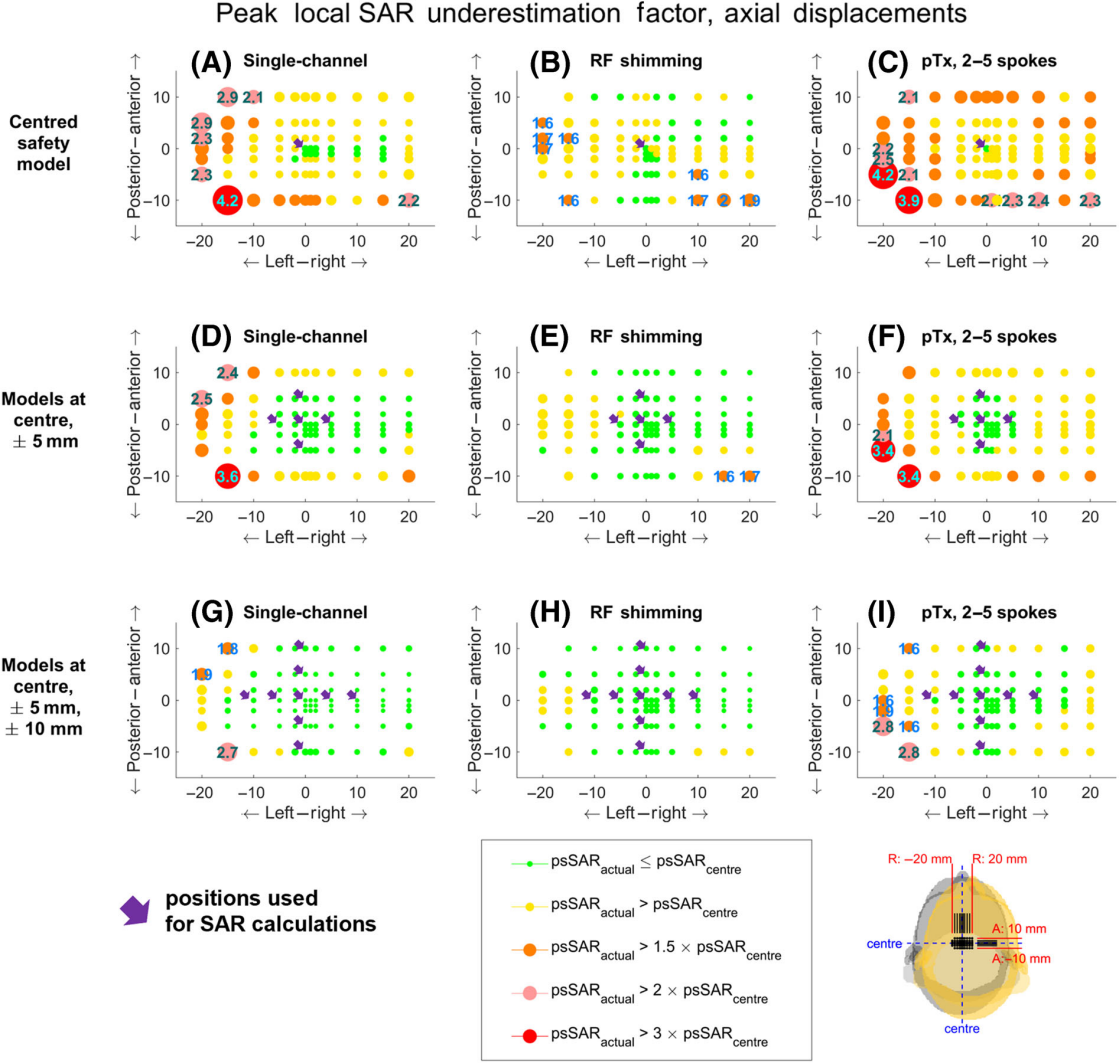
The positional variation of the peak local SAR underestimation in the axial plane is shown when the safety model consists of (A‐C) The centred body position, (D‐F) Five positions (centre, Right: ± 5 mm, Anterior: ±5 mm) and (G‐I) Nine positions (centre, Right: ±5 and ±10 mm, Anterior: ±5 and ±10 mm). The charts show maximum intensity projections along number of spokes and slices (where applicable). Left: Single‐channel mode. Middle: pTx mode, 1‐spoke pulse (RF shimming). Right: pTx mode, 2‐/3‐/4‐/5‐spoke pulses. The circles indicate the position of the centre of the head, similar to the inset in the bottom‐right corner. The size and colour of the circles indicate the SAR underestimation factor, with some values listed inside. psSAR, peak spatial value of local SAR; pTx, parallel‐transmit; RF, radiofrequency; SAR, specific absorption rate

The difference between the actual and estimated psSAR was most drastic for displacements in the axial plane. Figure 3 is a maximum intensity projection along spokes and slices, and it shows the largest ${\mathrm{SAR}}_{\text{actual}}/{\mathrm{SAR}}_{\text{safety}-\text{model}}$ ratio for all positions on the axial plane (rotations and other displacements are omitted here for conciseness because displacements in the axial plane yielded larger sensitivity). Figure 3 also shows the locations of the positions added to the safety model (indicated by small arrows). Connecting the outermost positions creates a square‐shaped region in which local SAR was never underestimated across the investigated pulses. While there are head positions immediately outside these squares that still showed an underestimation of SAR, this underestimation was confirmed to be less than 5% for the positions immediately adjacent to these regions.

As expected, SAR underestimation increases as the body gets closer to the coils. However, this behaviour was not monotonic or spatially smooth everywhere. This is because the pulse design algorithm was not constrained during spoke selection, and therefore the excitation k‐space trajectories can vary between adjacent head locations, leading to variations in SAR sensitivity to positional mismatch. Nevertheless, these local nonmonotonic variations were not substantial, as indicated by the relative size of the markers. Furthermore, the behaviour of ${\mathrm{SAR}}_{\text{actual}}/{\mathrm{SAR}}_{\text{centre}}$ is not symmetrical around the Cartesian axes. This is attributed to the imperfect centring of the head.

While positional variation‐related SAR underestimation increased with increased proximity to the coil elements, underestimation also depends on the channel weights and the interactions between coil elements. Even although L/P: 15/10 mm and L/P = 20/5 mm positions generally showed higher SAR underestimation, there was not a single position that always exhibited the worst‐case underestimation (Figure 3).

**FIGURE 4:**
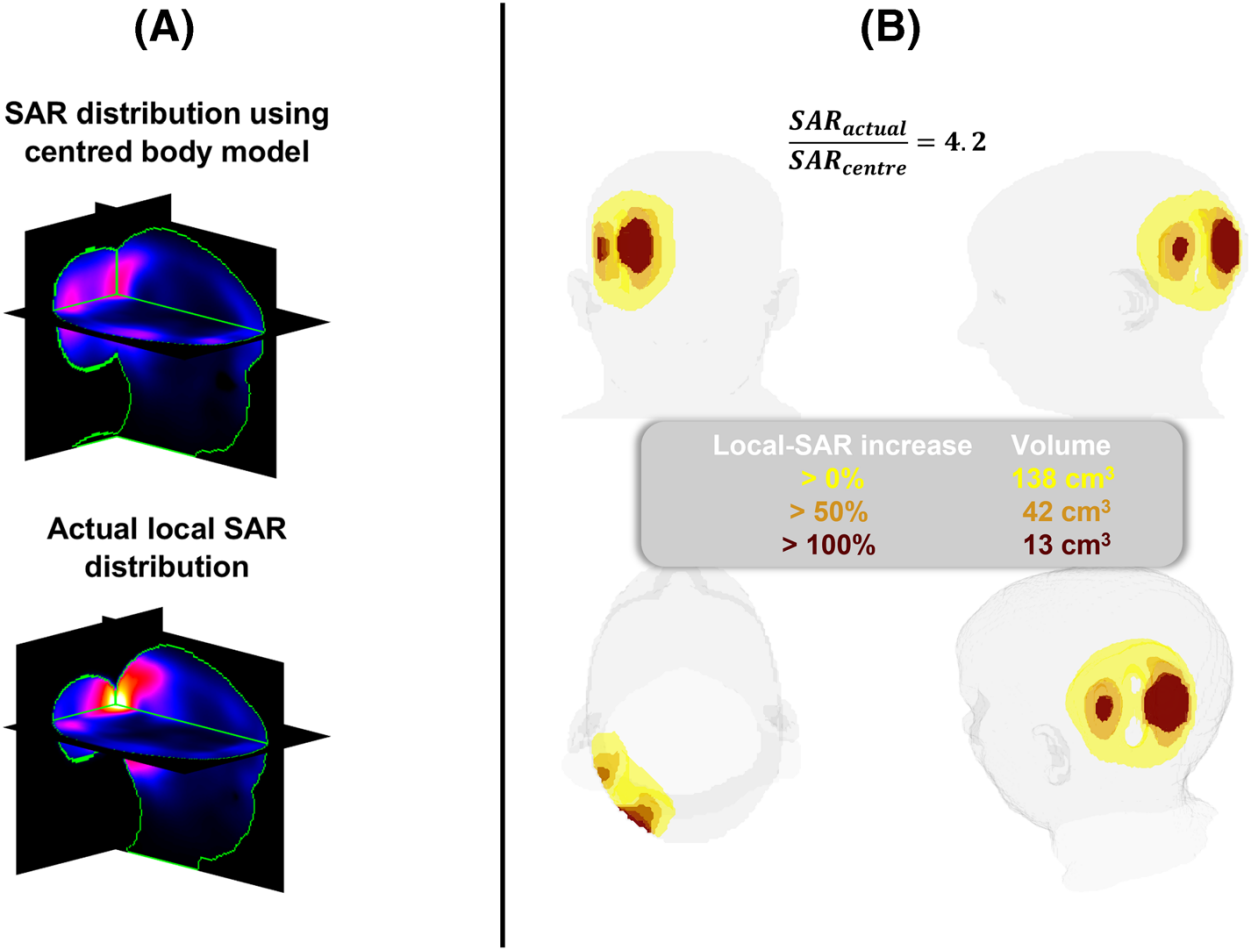
The region that was exposed to a higher level of local SAR than the peak estimated using the centred safety model is shown, for the 5‐spoke pulse designed for the Left: 15 mm, Posterior: 10 mm head position. (A) The location of the estimated psSAR and the actual psSAR. (B) The estimated psSAR was used as a threshold for the three‐dimensional actual local SAR distribution. The yellow colour indicates regions exposed to elevated levels of local SAR compared with the estimated peak, whereas orange and red colours indicate regions that received more than 50% and 100% higher local SAR compared with the estimated peak, respectively. psSAR, peak spatial value of local SAR; SAR, specific absorption rate

Figure 4 compares the three‐dimensional actual local SAR distribution for the worst‐case pTx pulse with the distribution and the psSAR estimated using the centred model. In the worst‐case scenario, the actual local SAR was 4.2‐fold as high as the estimated peak and a region of 13 cm^3^ was exposed to more than twice the estimated peak.

**FIGURE 5:**
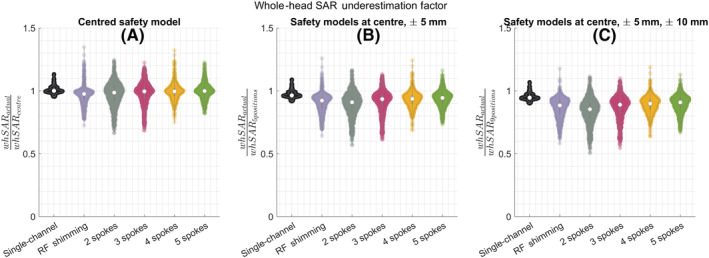
Violin plots show how the actual whSAR compares with the whSAR estimated using the centred model when the safety model consists of (A) The centred body position, (B) Five positions (centre, Right: ±5 mm, Anterior: ±5 mm) and (C) Nine positions (centre, Right: ±5 and ±10 mm, Anterior: ± 5 and ±10 mm). RF, radiofrequency; SAR, specific absorption rate; whSAR, whole‐head SAR

The effect of the positional mismatch was less pronounced for whSAR (Figure 5). In the worst cases, whSAR increased by 14% for the single‐channel mode and by 35% for pTx. Using more positions in the safety model reduced the underestimation factor to 10% (five positions) and 7% (nine positions) for single‐channel and to 26% (five positions) and 20% (nine positions) for pTx.

**FIGURE 6:**
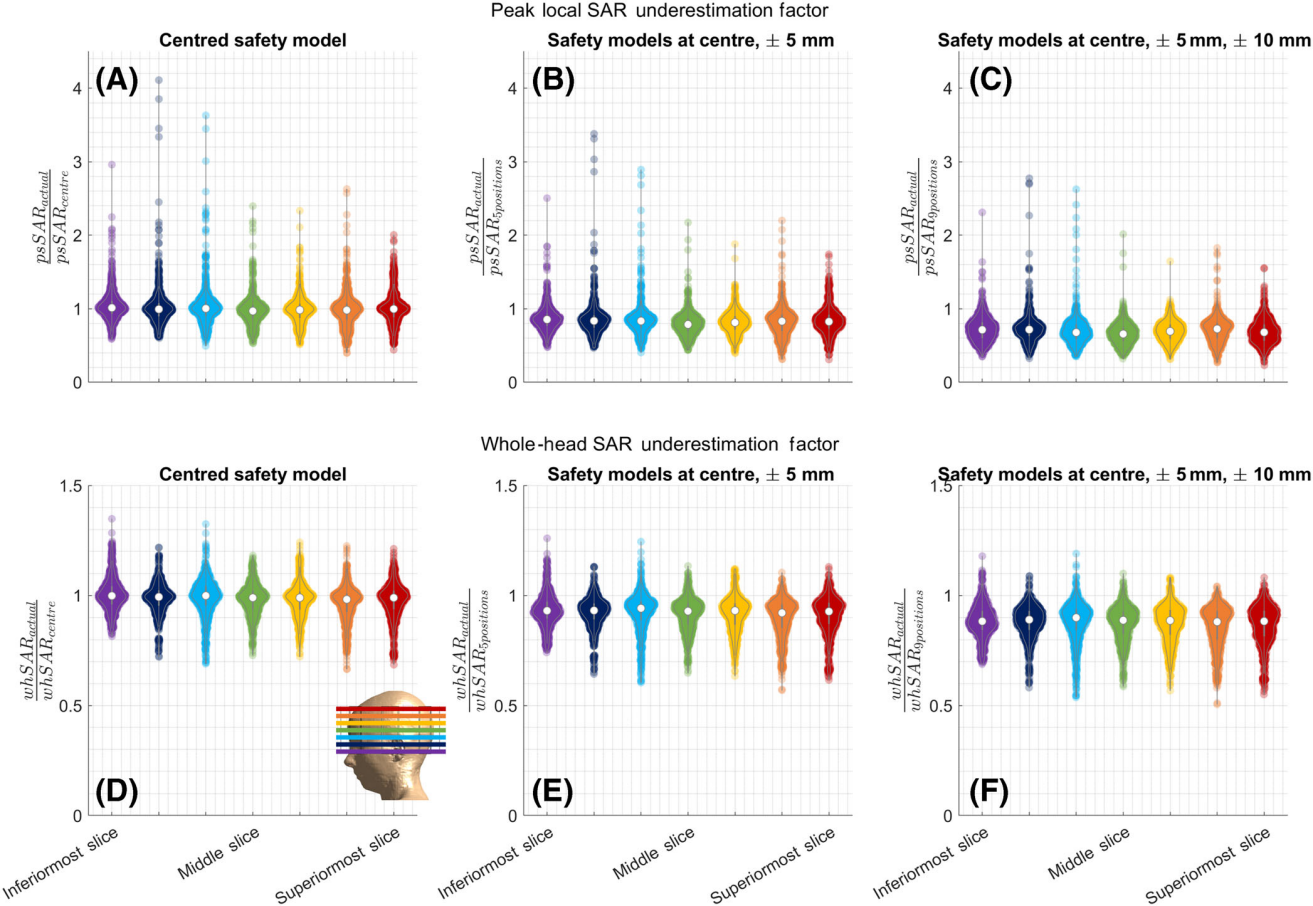
Violin plots show the SAR underestimation factors for (A)–(C) local and (D)–(F) whole‐head SAR for all pulses (single‐channel and parallel‐transmit with 1‐/2‐/3‐/4‐/5‐spokes) designed for imaging different slices, for all safety models. The safety model consists of (A) the centred body position, (B) 5 positions (centre, right: ±5 mm, anterior: ±5 mm), (C) 9 positions (centre, right: ±5 mm and ±10 mm, anterior: ±5 mm and ±10 mm). The colour coding of slices matches the colour coding of slice locations shown in the overlay. psSAR, peak spatial value of local SAR; SAR, specific absorption rate; whSAR, whole‐head SAR

The sensitivities of local and whole‐head SAR to positional mismatch were not specific to any part of the brain (Figure 6): 21/21 and 18/21 paired comparisons across slices (highest *p* value kept across spokes) and 9/10 and 8/10 comparisons across pulses (figure omitted) with different numbers of spokes (highest *p* value kept across slices) were insignificant for local and whole‐head SAR, respectively.

**FIGURE 7:**
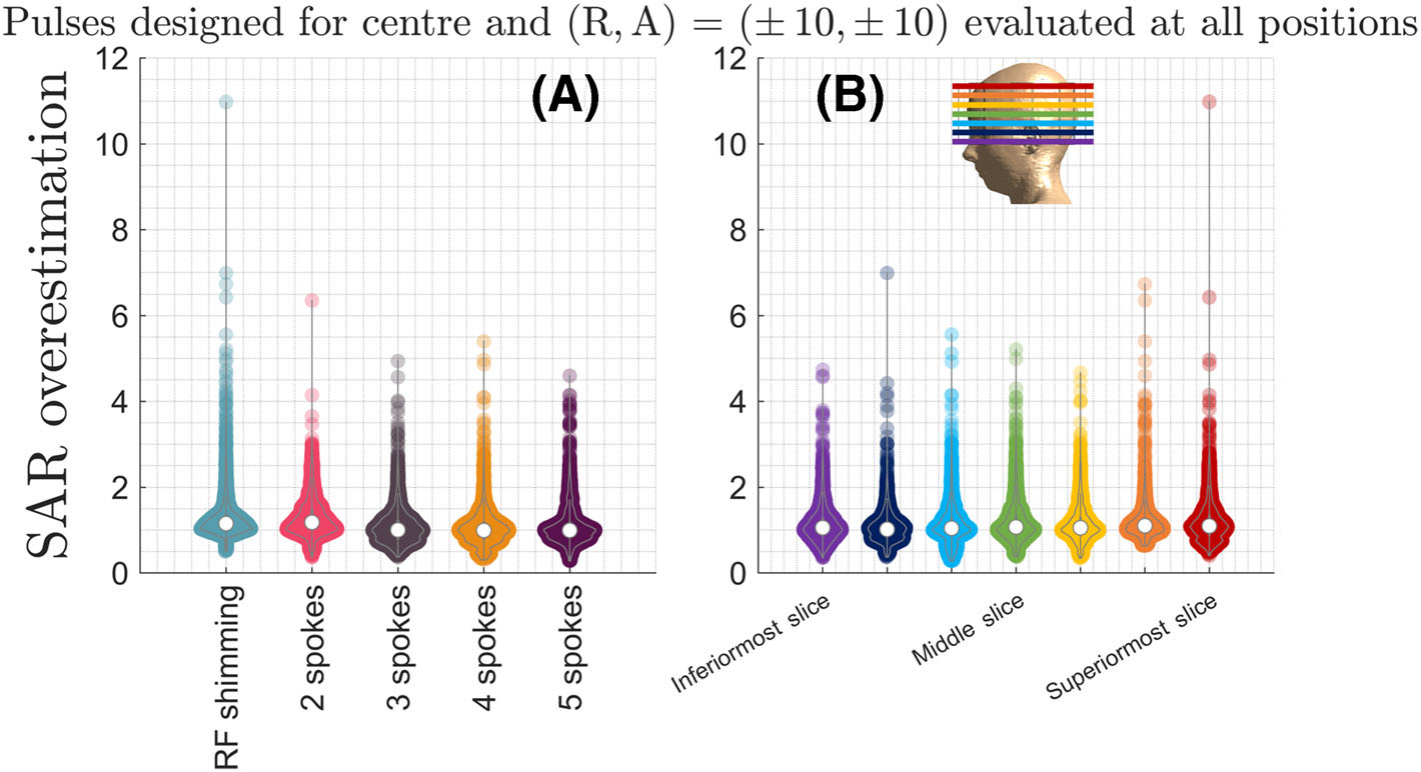
The local SAR overestimation caused by evaluating local SAR using all body positions for the 175 pulses designed for centre, and combinations of Right: ± 10 mm and Anterior: ± 10 mm is shown. Using all positions leads to a maximum SAR overestimation of 11‐fold for RF shimming. The variation of the overestimation is shown for (A) Single‐channel and pTx pulses with different number of spokes (for all slices) and (B) For different slices (for all pulse types). The colour coding of slices matches the colour coding of slice locations shown in the overlay. pTx, parallel‐transmit; RF, radiofrequency; SAR, specific absorption rate

**FIGURE 8:**
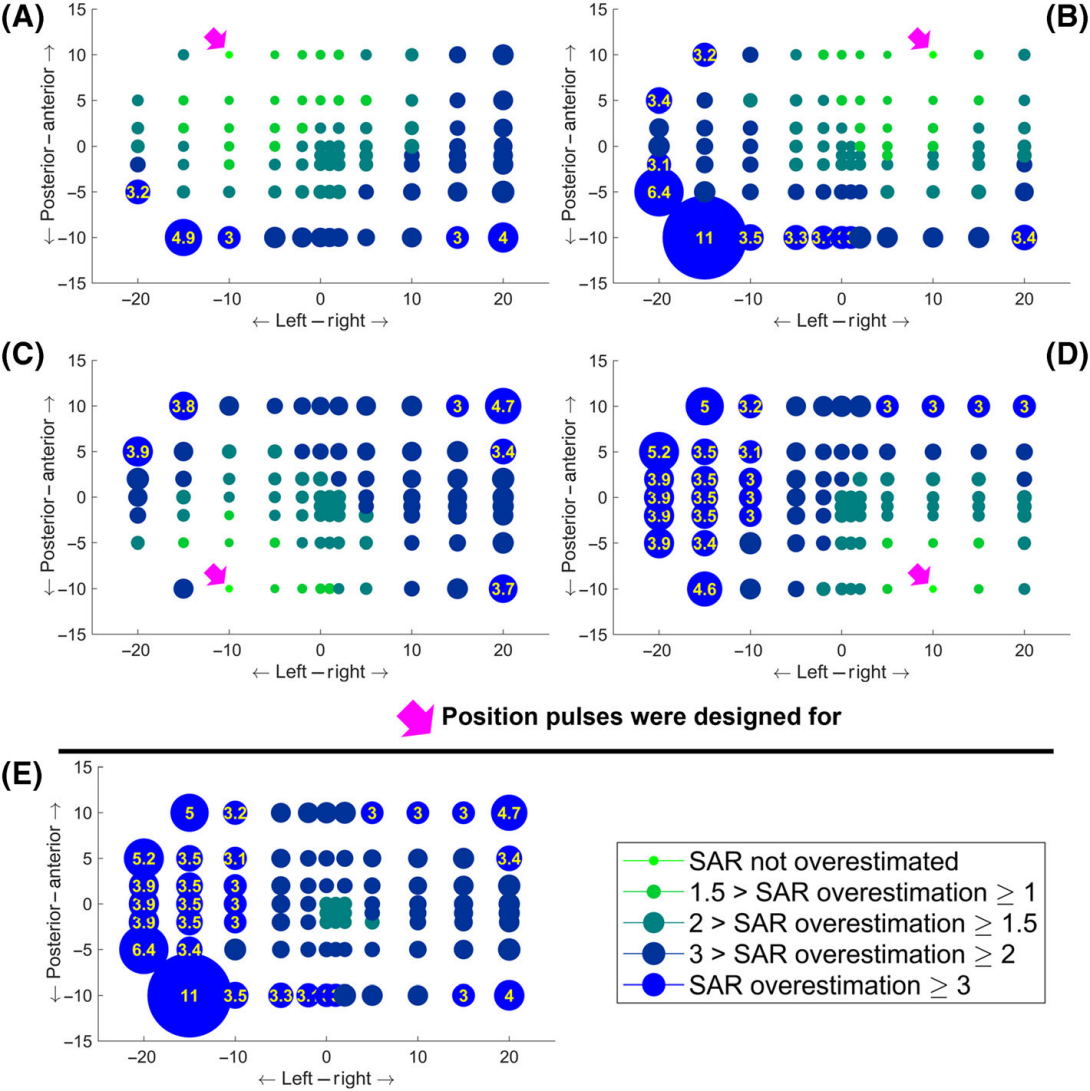
The local SAR overestimation caused by including different body positions in the safety model are shown for positions on the axial plane, for the 175 pulses designed for centre, and combinations of Right: ± 10 mm and Anterior: ± 10 mm. (A‐D) SAR overestimation increases as the body positions used in the safety model get further away from the actual position the pulses were designed for (indicated by the pink arrows). (E) The worst‐case overestimation caused by including the body model at each position in the safety model across all 175 pulses. The size and colour of the circles indicate the overestimation factor, with some values listed inside. SAR, specific absorption rate

Figures 7 and 8 show SAR overestimation when all positions are used in SAR calculations for pulses designed for centre and four off‐centre positions. The highest psSAR overestimation was for RF shimming at 11‐fold, with more than 4‐fold overestimation being observed for all pulse types (Figure 7A), all slices (Figure 7B) and multiple positions (Figure 8E). Figure 8A‐D show that, as expected, SAR overestimation increases as the body position used for SAR estimation gets further away from the position the pulse was designed for.

**FIGURE 9:**
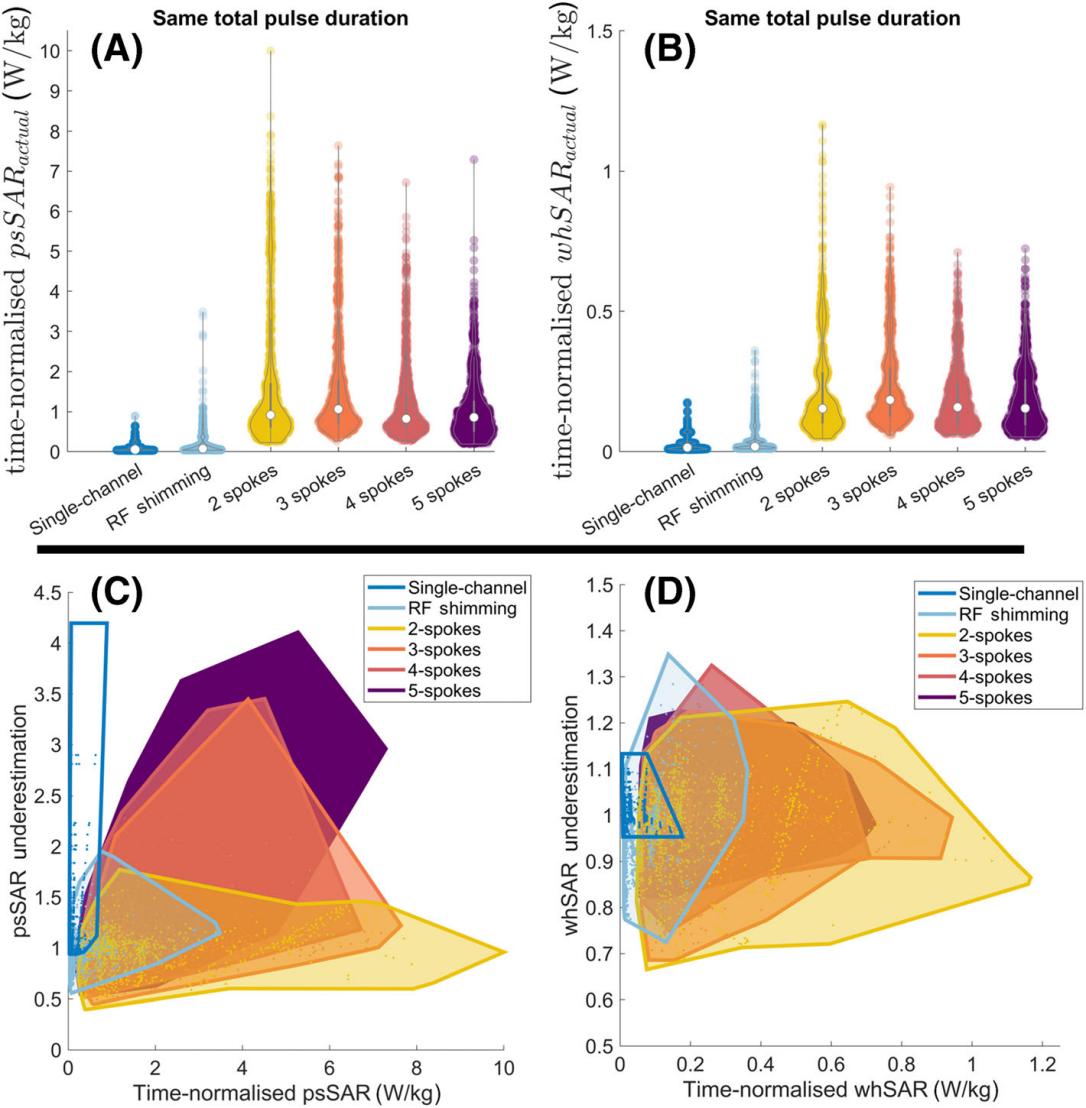
(A and B) Actual peak local SAR and whSAR values for all pulses. SAR values were time‐normalised, that is, the same pulse duration was used for all pulses, regardless of the number of spokes. The common pulse duration was set so that all pulses would be under the safety limits. (C and D) The actual psSAR and whSAR values are shown against the sensitivity of these metrics to positional mismatch. Shaded regions enclose all datapoints for that excitation mode. psSAR, peak spatial value of local SAR; SAR, specific absorption rate; whSAR, whole‐head SAR

Figure 9 shows time‐normalised actual psSAR and whSAR values for all pulses, for the same TR and flip angle. Single‐channel and RF shimming offered reduced maximum and average SAR values compared with 2–5 spoke pTx pulses (parts (a) and (b)). Parts (c) and (d) plot the positional sensitivity of SAR with respect to actual SAR values: 3–5 spoke pulses yielded high SAR and high positional sensitivity. RF shimming offered a tradeoff between single‐channel and 2‐spoke pTx, with lower positional sensitivity and lower psSAR values.

**FIGURE 10:**
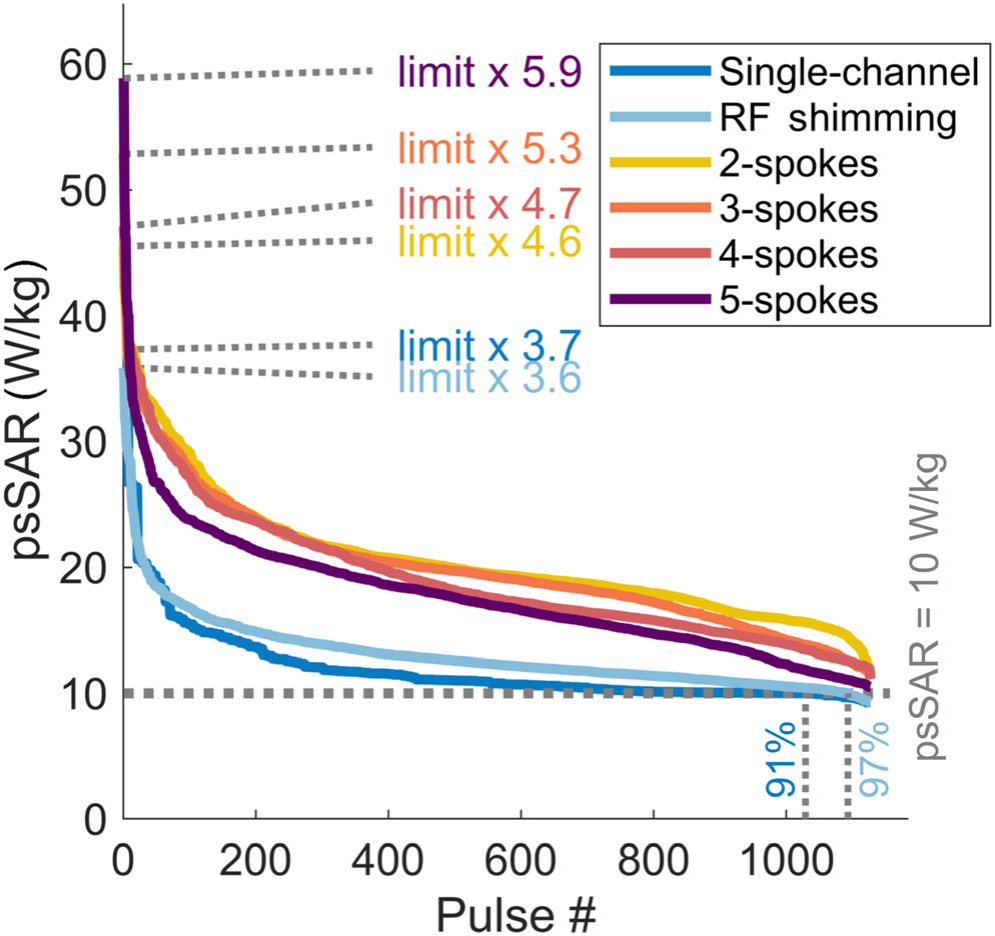
Actual peak local SAR (psSAR) is shown for all pulses when the actual whole‐head SAR (whSAR) for each pulse was set to 3.2 W/kg (by changing individual pulse durations). The psSAR values for pulses designed for different slices and positions for each excitation strategy were ordered from highest to lowest. The maximum psSAR is shown next to the vertical axis and the percentage of pulses that exceed 10 W/kg when whSAR = 3.2 W/kg is shown above the horizontal axis for each case (100% for 2–5 spokes, omitted). RF, radiofrequency; SAR, specific absorption rate

The investigated coil structure coincidentally (i.e., not by design) yielded 10 W/kg peak local SAR when whSAR was 3.2 W/kg at the centred position in the single‐channel mode (same flip angle, minimum TR for all pulses). Because the local SAR was more sensitive to positional variations, psSAR exceeded 10 W/kg for 91% of the positions when the actual whSAR was maintained at 3.2 W/kg, reaching a maximum of 37.3 W/kg in the single‐channel mode (Figure 10). For RF shimming, the maximum psSAR was 35.9 W/kg and psSAR exceeded 10 W/kg in 97% of cases. Multispoke pulses led to higher psSAR than single‐channel and RF shimming; for 2–5 spokes, local SAR exceeded 10 W/kg in all cases, reaching a maximum of 58.9 W/kg. The reported values are for when whSAR was set to 3.2 W/kg at each position rather than at the centre. For a minority of cases (9% for single‐channel, 3% for RF shimming), whSAR exceeded its limit when psSAR was set to 10 W/kg.

**FIGURE 11:**
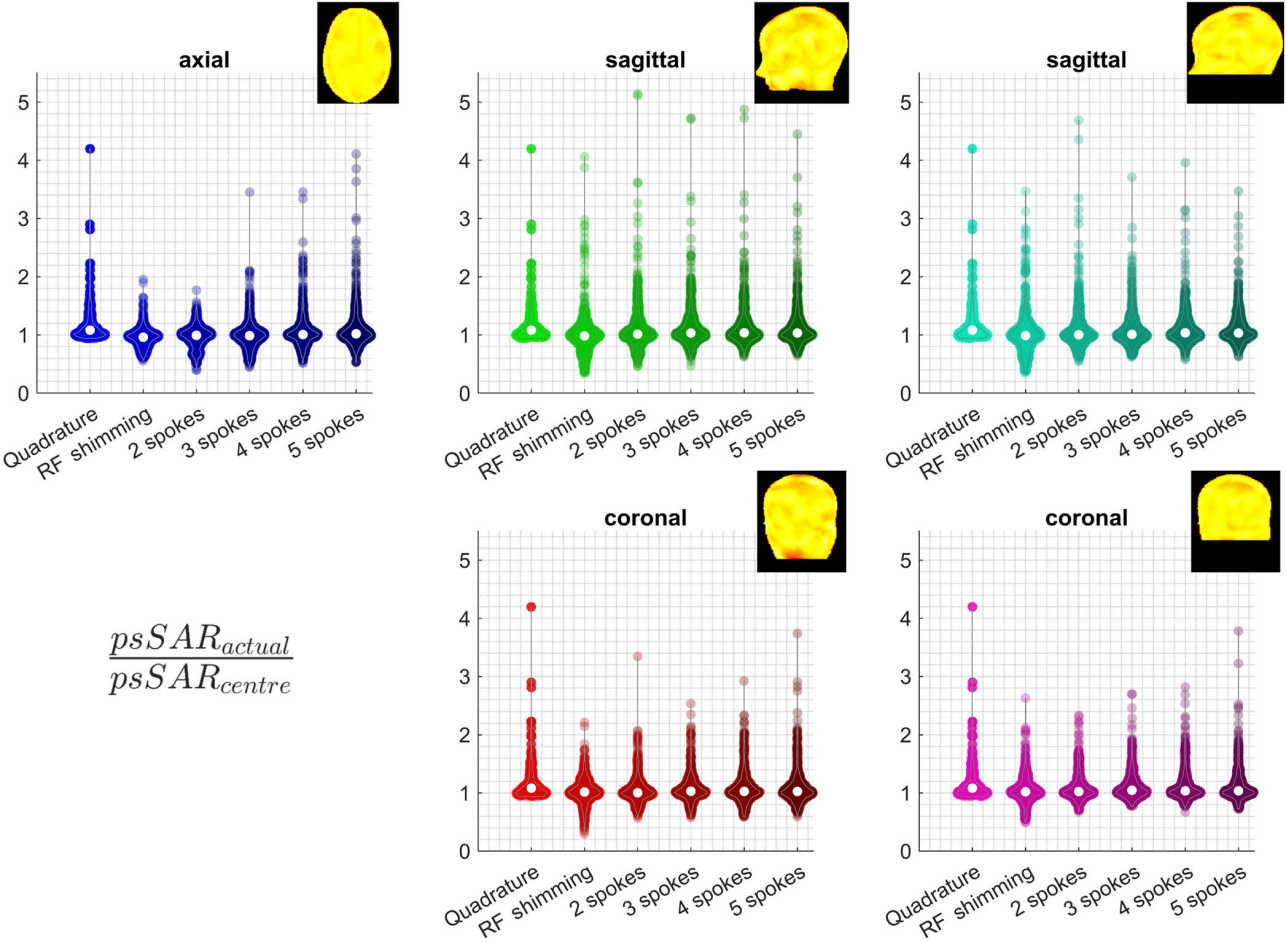
Violin plots show the underestimation factor of the peak local SAR when the safety model is at the centred body position, for different slice orientations and ROIs. The axial results in Figure 2A are duplicated here for better comparison. Example excitation profiles indicate ROI definitions. While axial RF shimming and 2‐spoke pulses yield reduced SAR sensitivity to positional mismatch, coronal and sagittal slices show considerable SAR sensitivity for all pulse types. psSAR, peak spatial value of local SAR; RF, radiofrequency; ROI, region of interest; SAR, specific absorption rate

**FIGURE 12:**
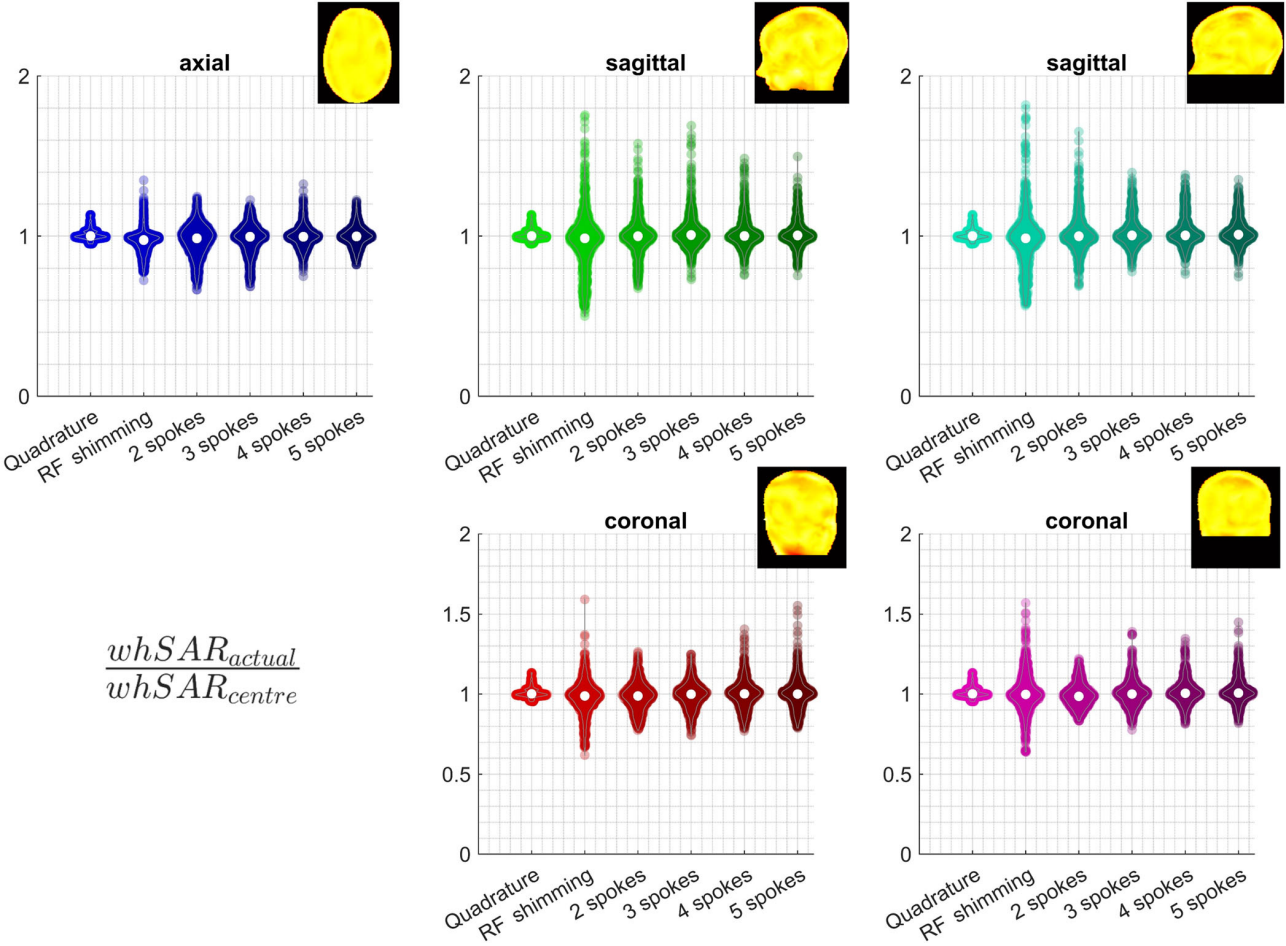
Violin plots show the underestimation factor of whSAR when the safety model is at the centred body position, for different slice orientations and ROIs. The axial results in Figure 5A are duplicated here for better comparison. Example excitation profiles indicate ROI definitions. Sagittal and coronal excitation led to higher positional sensitivity for whSAR. While SAR underestimation was less than 40% for axial slices, it was as high as 80% and 60% for sagittal and coronal slices, respectively. RF, radiofrequency; ROI, region of interest; SAR, specific absorption rate; whSAR, whole‐head SAR

Figures 11 and 12 demonstrate SAR underestimation when a single centred body model is used, for pulses designed for different slice orientations. Sagittal excitation pulses showed a higher sensitivity to positional mismatches than axial and coronal pulses. The highest psSAR underestimation factors for pTx pulses were 5.2‐, 4.2‐ and 3.8‐fold for sagittal, axial and coronal pulses, respectively (Figure 11). While RF shimming and 2‐spoke pulses demonstrated lower sensitivity to positional mismatch for axial pulses, similar behaviour was not observed for coronal or sagittal pulses. Neither ROI demonstrated a clear benefit over the other for sagittal and coronal imaging.

Sagittal and coronal pulses showed a higher sensitivity to positional mismatch than axial pulses, for whSAR (Figure 12). The worst‐case whSAR underestimation was 1.8‐fold for sagittal, 1.6‐fold for coronal and 1.4‐fold for axial pulses. Similar to psSAR, the ROI definition affected SAR underestimation, but did not show beneficial behaviour that can be exploited for reduced SAR sensitivity in either case.

### 3.1 Variation of patient head position within commercially available head coils

For the imaging data acquired at CUBRIC, maximum variations of 25/14/10/30 mm were observed for the leftmost/rightmost/posteriormost/anteriormost extent of the head across the 66 positions within the pTx coil, while the values were 22/24/6/28 mm for the single‐channel coil, respectively. The investigation of the seven publicly available datasets revealed a maximum variation between extreme cases of 33/29/42/50 mm for the leftmost/rightmost/posteriormost/anteriormost edges of the head, respectively. The centre of the head varied by 26 mm on the right–left axis and by 36 mm on the anterior–posterior axis.

## 4 DISCUSSION

Patient‐specific pTx pulses are inherently position‐dependent as position‐specific B_1_^+^‐maps are used for pulse design. However, safety models on MRI scanners are fixed (i.e., not updated according to current patient position). This creates a positional mismatch between what is used for pulse design and what is used to estimate/constrain the pulse's safety aspects. This study investigated the effect of this positional mismatch on local and whole‐head SAR. For this purpose, a virtual body model was simulated at 161 positions inside an eight‐channel transmit array. For each position, single‐channel (quadrature excitation), RF shimming (1‐spoke pTx) and multispoke pTx pulses were designed to achieve a homogeneous excitation. Axial, sagittal and coronal imaging were investigated. Actual local and whole‐head SAR were compared with values calculated using safety models that consist of body models at the centred position, and five and nine body positions on the axial plane. Local SAR of 175 pulses designed for five different locations was evaluated at all off‐centre positions to investigate how much SAR is overestimated when all positions are used.

Initial patient position has a considerable impact on local SAR for both single‐channel and pTx modes. For both modes, peak local SAR was underestimated by up to 5.2‐fold using a centred safety model. Local SAR shows significant dependence on the tissue‐coil proximity, highlighted by the sensitivity of local SAR to positional mismatch for single‐channel transmit (for which the channel coefficients are fixed in relation to each other). The variation of local SAR with coil‐tissue proximity is in agreement with Deniz et al.,^65^ in which considerable increases in global and local SAR were shown as the distance between the coil elements and a homogeneous phantom decreased. Positional mismatch may cause misrepresentation of the head‐coil proximity and lead to SAR underestimation. Furthermore, it may cause incorrect estimation of the locations of regions with elevated SAR, which may cause concerning hotspot overlaps if SAR hopping^66^ is used.

Importantly, certain pTx modes are more robust to positional uncertainties. RF shimming and 2‐spoke pulses yielded more than 50% lower positional sensitivity compared with both single‐channel and 3‐/4‐/5‐spoke pTx pulses for axial imaging. This is attributed to the self‐correcting nature of RF shimming, that is, when the head is off‐centre, the coil elements closer to the head create higher electric and magnetic fields compared with the centred position. RF shimming reduces the channel weights for these coils to achieve the target flip angle, also indirectly reducing local SAR, which is referred to as self‐correction in this paper. Similar behaviour was observed for 2‐spoke pulses, potentially because degrees‐of‐freedom were limited as the first spoke was prescribed to pass through the centre of k‐space. With both spokes freely placed, SAR underestimation behaviour for 2‐spoke pulses might be similar to that displayed by 3‐spoke pulses.

The robustness of RF shimming to SAR underestimation (observed for axial imaging) does not extend to more spokes. Because channel weights for all spokes are reoptimised when a new spoke is selected, each spoke does not necessarily target a homogeneous flip angle distribution; but instead the spokes target complementarily inhomogeneous distributions to improve the overall flip angle. Some of these distributions may yield large weights for the coil elements close to the body, increasing the sensitivity of local SAR to the patient position. Pulses with more time points (e.g., those using spiral trajectories) may behave more similarly to 5‐spoke pulses than RF shimming and yield high sensitivity to positional mismatch.

The sensitivity of SAR to positional mismatch, and the robustness of RF shimming thereof, are heavily influenced by slice orientation. While axial RF shimming and 2‐spoke pTx pulses demonstrated increased robustness against positional mismatch‐related SAR underestimation, similar behaviour was not observed for sagittal and coronal imaging. This is attributed to the coil structure investigated in this study. Because the coil elements are distributed azimuthally, they demonstrate high excitation efficiency in comparatively similarly sized and rather distinct regions in the axial plane, and higher independence from each other. In sagittal/coronal planes, a subset of coils yield much higher excitation efficiency compared with the other elements, and the field distributions of some coils show higher levels of linear dependence. Hence, RF shimming may still need to rely on the subset of more‐efficient channels, even if those are closer to the body (and therefore lead to elevated local SAR), and therefore may not exhibit self‐correction, as previously mentioned. However, this is not necessarily a limitation for certain slice directions, as coil elements distributed to provide better control in sagittal/coronal/oblique slices, such as multirow arrays, may lead to increased degrees of self‐correction.

Whole‐head SAR was not as sensitive to the position of the head as peak local SAR. Although whSAR only increased by 14% for single‐channel mode, the worst‐case increase was much higher for pTx at 80%. Nevertheless, the reduced sensitivity to positional variations compared with local SAR is attributed to whSAR being averaged over a larger volume. When the head gets closer to some coil elements, it is getting further from the other coils, and the increase in power absorbed in one side of the head is balanced by the decreasing power absorption on the other side.

For most (axially selective) pulses investigated, local SAR exceeded safety guidelines before whSAR did. The coil structure coincidentally (i.e., not by design) yielded psSAR = 10 W/kg and whSAR = 3.2 W/kg when used in single‐channel mode at the centred position. For a different coil structure that yields a more homogeneous local SAR distribution, whSAR may exceed limits before local SAR does for more pulses. Hence, one metric may not necessarily substitute for the other and both metrics might need to be used to ensure safety depending on the coil structure.

A better understanding of the contents of the coil safety models that are distributed with coils or scanners is needed. Such models can range from a single body model simulated at the centre to multiple body models at multiple positions/orientations. Using a centred safety model yielded a considerable underestimation of SAR (up to 4.2‐fold for axial imaging). Adding more positions to the safety model reduced the overall underestimation and created a region in which SAR was never underestimated. This is attributed to the body models at surrounding positions being closer to the coil elements. Notwithstanding the interactions between fields from multiple channels being nontrivial, the body at the position closer to the coil elements (the interaction of which create the very high local SAR values) observes an increase in the local SAR because of increased proximity. Nevertheless, this behaviour can fail in certain cases, depending on interactions between coils, and using only the extreme body positions in the safety model may still lead to SAR underestimation. To ensure patient safety, more clarity is needed in the communication from scanner vendors and coil manufacturers to end‐users regarding how the safety models were generated and what range of cases and safety margins were incorporated.

Using all possible body positions limits imaging performance considerably, especially for self‐correcting modes like RF shimming. Using more positions in the safety model reduces underestimation, but it also increases overestimation (as expected), and overestimation increases more rapidly than underestimation reduces. Note that underestimation and overestimation are not multiplicative inverses because of the maximum and minimum projections along pulses and body positions included in the safety model. Adding an irrelevant position far from the actual position into the safety model might not reduce underestimation but could considerably increase overestimation. Here, using all positions in the safety model led to an 11‐fold overestimation of local SAR for RF shimming with more than 4‐fold overestimation observed for all pulse types.

Both local and whole‐head SAR were more sensitive to positional mismatch on the axial plane compared with rotations and superior–inferior displacements. Here, rotation was around the centre of the head rather than the neck. The latter may bring the head much closer to the coil elements for the same rotation, leading to higher variations. No specific slice location was identified that affected the positional sensitivity of local and whole‐head SAR.

The electromagnetic simulations and data preprocessing performed were consistent with Kopanoglu et al.^14^ Coil matching and tuning were performed for only the centred body model. The input power in each channel was normalised to 1 W, neglecting the reflections at the feed port–coil interface and thereby imitating a patient‐specific prescan calibration that adjusts for reflections at the ports. Without Tikhonov regularisation, pulse design would lead to the same normalisation to achieve the target flip angle, even in the absence of a prescan calibration. However, with Tikhonov regularisation of RF power in each channel, this normalisation can have an effect on channel coefficients, if both conditions are satisfied: (i) the normalisation factor varies considerably across channels and positions; and (ii) a highly restrictive power regularisation is used. In this study, neither of these conditions were satisfied, as the normalisation factor varied by less than 8% across the coils for the centre location and the location with the largest displacement (R: 20/A: 10 mm), and the power regularisation was used to prevent unrealistic power levels without being overly restrictive. The input power includes dissipation in the lumped elements, radiated power, coupling to other coils and the power absorbed in the body. The reflections at the coil–port interface were overridden only for coil‐feeding purposes and were included in coil‐coupling calculations. The effect of positional variations on coil loading, matching, tuning and coupling were inherently incorporated in the results. These variations may affect electromagnetic field distributions (and therefore, SAR) and may be different for other coil designs (i.e., microstrip designs, antenna elements, shielded coils, overlapping loops).

The results show similarities with the literature. While Murbach et al. observed much higher sensitivity of local SAR to position along the axis of the bore for single‐channel at 1.5 T^33^, ^34^ and two‐channel RF shimming at 3 T,^35^, ^36^ the positional variations were more extreme as the focus was on imaging different body parts. Here, results indicated less sensitivity to superior–inferior displacements compared with axial, in agreement with Shajan et al.,^39^ which reported less than 10% variation in local SAR because of superior–inferior displacements. Also, 2.1‐ and 2.25‐fold increases in psSAR were reported in recent studies^40^, ^41^ attributed to superior–inferior displacements with less pronounced variations along right–left and anterior–posterior.^41^ Such differences are not unexpected because those studies^40^, ^41^ investigated neonatal models inside a larger transmit coil, reducing coil‐tissue proximity in the axial plane. Le Garrec et al.^28^ reported a probabilistic analysis of local SAR variations and discussed that a safety margin of 1.5 is unlikely to be exceeded for anterior–posterior and superior–inferior displacements. Here, higher increases in local SAR were observed for off‐axis axial displacements, possibly because this type of motion further increases coil‐tissue proximity. Variations of up to 150% in psSAR for displacements of 12.5 mm in the anterior–posterior for quadrature excitation and RF shimming with a four‐channel pTx coil have been reported,^37^, ^38^ similar to the values reported here. Kopanoglu et al.^14^ focused on motion‐related effects on SAR and reported up to a 3.1‐fold increase in psSAR. Plumley et al.^32^ expanded on that study^14^ and investigated the effect of altering coil dimensions on the motion sensitivity of local SAR. Further to translational/rotational position of the body models, partial positional variations (i.e., position of the limbs relative to each other and to the body) can also have a profound effect on local SAR.^67^, ^68^ A recent study has demonstrated that using personalised SAR models can reduce the effect of the differences between the simulated models and the actual subject on SAR underestimation.^69^ Such models would inherently be patient‐position aware and, therefore, can potentially be used to overcome positional variation‐related SAR underestimation, especially if the associated ~30‐min workflow for pTx observed in the initial implementation^69^ can be reduced to clinically feasible times. Simultaneous multislice imaging has been shown to reduce the effect of patient motion on SAR underestimation.^70^ A future study may investigate whether a similar approach may also reduce SAR underestimation due to positional variations as well.

The current study shares some characteristics with the one conducted by Kopanoglu et al.,^14^ including parts of the dataset (and corresponding simulation techniques) and the pulse design method. However, the cases studied are distinct and complementary. This study investigates safety concerns caused by positional differences between the actual initial patient position and the safety model (i.e., the effect of a nonideal safety model). Furthermore, this study concerns stationary patients. On the other hand, Kopanoglu et al.^14^ assumed an ideal safety model and investigated the effect of within‐scan patient motion that occurs after initial patient positioning. In the earlier study,^14^ the B_1_^+^‐maps and the electric fields used for pulse design and safety estimations, respectively, matched each other position‐wise. Then variation in SAR was calculated in the presence of patient motion using off‐centre Q‐matrices for pulses designed using centred B_1_^+^‐maps. The current study, on the other hand, takes a step back and questions the validity of SAR calculations if the initial patient position does not match the safety models. In this case there is a positional mismatch between the B_1_^+^‐maps and the electric fields used for pulse design and safety estimations, respectively. Hence, the studied cases do not overlap, and also they are not mutually exclusive. In fact, in practice, depending on the scanner safety model (few positions vs. all positions), the implications investigated here (single vs. multiple position cases) would coexist with those in the earlier study^14^ in the presence of within‐scan patient motion. As an example, consider safety models consisting of simulation(s) at position(s) A, initial patient positioning at position B, the patient moving during the scan to position C. In this case, the two effects may either mitigate or exacerbate SAR concerns. But such an investigation is beyond the scope of the current study. In another specific scenario, if there is patient motion but the effects of motion on B_1_^+^‐maps are estimated^71^ and corrected in real time,^52^ the earlier study^14^ can morph into the current study.

The differences between the current and the earlier study^14^ can also be observed in the uncovered effect of pulse types on (local and whole‐head) SAR sensitivity for axial imaging. In Kopanoglu et al.,^14^ SAR sensitivity to patient motion was lowest for single‐channel transmit, was slightly higher for RF shimming, peaked for 2‐/3‐spoke pulses, and was reduced for 4‐/5‐spoke pulses. Here, the SAR sensitivity to positional mismatch was similar for single‐channel and 3‐/4‐/5‐spoke pTx pulses and considerably lower for RF shimming and 2‐spoke pulses with RF shimming offering lower SAR levels. Hence, RF shimming offers a balance between SAR, positional sensitivity and motion sensitivity.

This study has limitations. The dataset is not comprehensive as certain combinations of displacements/rotations were not investigated. In practice, coil dimensions vary across scanners and field strengths, and have a direct effect on how much positional variation can be observed across participants. Maximum variations of 30 and 28 mm were observed for the scans conducted at CUBRIC with a pTx array and a single‐channel transmit coil, respectively, whereas a 50 mm maximum variation (anterior) was calculated when seven publicly available datasets were analysed. The diameter of the coil structure affects SAR underestimation and overestimation as it affects coil‐tissue proximity. Therefore, the numerical results cannot be extrapolated to other coil structures. With a larger coil, the SAR variation for the same absolute displacement (e.g., 10 mm) might be less.^32^ In practice, the maximum proximity of the head to the transmit coil elements would be limited because of the coil housing. Furthermore, the presence of a receive‐array within the transmit‐array would further limit the maximum coil‐tissue proximity. Nevertheless, coil‐tissue proximity can be high when tx/rx arrays (i.e., transmit/receive arrays that utilise the same coil elements for both excitation and acquisition) are used. Other virtual body models could have affected the results, similar to Ipek et al.^29^ For example, the positional displacements for a larger body model such as Duke would be more limited and the baseline SAR could have been higher because of the higher filling ratio of the coil. While the numerical values might differ, the tendencies of increasing SAR with increasing coil‐tissue proximity^65^ and increasing overestimation with increasing actual position‐model distance are expected to be similar for other coils and other body models. In Plumley et al.,^72^ different body models were investigated in terms of their SAR sensitivity to patient motion as an extension to Kopanoglu et al.,^14^ and the worst‐case sensitivity of SAR to patient motion was similar across body models.

## 5 CONCLUSIONS

It is important to have clearer communication, from manufacturers to users, regarding what imaging scenarios the safety models represent. While safety models that include all possible patient positions may ensure safety, they may be extremely over‐restrictive on imaging performance, especially for self‐correcting modes such as RF shimming. Alternatively, when a nonexhaustive set of positions is used in the safety model, differences between safety‐model positions and actual patient positions can cause substantial variations between estimated and actual local SAR and less pronounced variations in whSAR. To minimise such variations, patients should be positioned as close to the positions represented in the safety model as possible. In the absence of such information, patients should be positioned as equidistantly from the coil elements as possible (for similar coil elements), especially in applications constrained by local SAR. In such cases, mechanical means (e.g., padding and inflatable cushions) can also be used to ensure against the patient being positioned too close to the coil elements. RF shimming offers much reduced positional sensitivity of SAR because of its self‐correcting nature and can be the preferred mode of operation. Imaging mode and position‐dependent safety models would facilitate higher performance imaging while ensuring safety.
